# Click Access to a Cyclodextrin-Based Spatially Confined AIE Material for Hydrogenase Recognition

**DOI:** 10.3390/s18041134

**Published:** 2018-04-08

**Authors:** Rui Zhao, Bin Li, Yong Wang, Wenping Hu

**Affiliations:** 1 Tianjin Key Laboratory of Molecular Optoelectronic Science, Department of Chemistry, School of Science, Tianjin University, Tianjin 300072, China; zhaoruitju163@163.com (R.Z.); huwp@tju.edu.cn (W.H.); 2 School of Chemical Engineering and Technology, Tianjin University, Tianjin 300072, China; libin@tju.edu.cn; 3 Collaborative Innovation Center of Chemical Science and Engineering (Tianjin), Tianjin 300072, China

**Keywords:** click chemistry, cyclodextrin, aggregation induced emission, sensing, hydrogenases

## Abstract

The spatial confinement of conjugated phenyl rotators is a compulsory requirement for the fluorescence enhancement of aggregation induced emission (AIE) molecules. This work reports a novel spatially confined AIE material by restricting several tetraphenylethylene (TPE) molecules around the primary face of β-cyclodextrin (CD) via a Cu(I) catalytic 1,3-dipolar cycloaddition reaction (click chemistry). The spatial confinement effect was found to significantly enhance the fluorescence emission when compared with a single TPE modified CD. In addition, the emission maxima took place with the dimethyl sulfoxide volume ratio of 30% in a water mixture, which is remarkably different from traditional AIE molecules. Benefiting from the CD’s complexation effect, this material exhibits a selective fluorescence quenching property in certain hydrogenases and can be used as a fluorescence probe for hydrogenase sensing. This demonstrates the potential of the spatially confined AIECD for practical applications.

## 1. Introduction

In contrast to the traditional aggregation caused quenching (ACQ) [1], Tang et al. (2011) reported an interesting turn-on fluorescence behavior and named it the aggregation induced emission (AIE) phenomenon. Fluorophores with AIE properties usually have no fluorescence in their dilute solutions, because of the non-radiative decay caused by intramolecular motions. However, they become highly emissive in aggregate states caused by the restricted intramolecular rotation (RIR) mechanism [2,3]. Nowadays, the investigation of AIE molecules has become popular in the field of advanced functional materials, because of their distinctive fluorescence property. A series of chemical and biological sensors have been developed and applied based on a turn-on mechanism. However, because of the uncontrollable aggregation process, their fluorescence emission always exhibits a bathochromic-shift or unstable emission intensity, which is a negative factor for sensing usage. Hence, the construction of a novel highly fluorescence material—by confining AIE molecules [4,5] into microenvironments and via fluorescence quenching—is a good alternative for solving the above issues.

As the second generation of supramolecular compounds, cyclodextrins (CDs) are known to embody a unique hydrophobic inner cavity and a hydrophilic surface—originating from the hydroxyl moieties on the rims [6,7]. CDs show an exceptional ability to selectively bind suitably-sized aliphatic and aromatic molecules to form inclusion complexes [8,9]. This demonstrates great potential in various applications, such as sensing, catalysis, separation, adsorption, and the removal of organic pollutants from contaminated water [10]. Another interesting characteristic of CDs is their nano-sized molecular skeletons, which makes them favorable for applications in drug delivery [11] and nano-machines. Since the diameter of the β-CD cavity is about 0.5 nm and the seven hydroxyl moieties of the primary face are easily modified, β-CD can provide an excellent platform for spatially confining AIE molecules. 

On a separate note, hydrogenases can catalyze the reversible oxidation of H_2_ and the reduction of protons and they have since attracted interest from many researchers [12,13]. According to the structures of active sites, hydrogenases are classified as [NiFe]-hydrogenases, [FeFe]-hydrogenases, and [Fe]-hydrogenases [14]. Currently, we are facing the dual pressures of limited fossil fuels and increasingly serious environmental problems. Hydrogen is a renewable energy source—which is characterized as being clean, efficient, pollution-free, and environmentally friendly, along with many other advantages [15]—and is considered to be one of the most promising new energy sources [16,17]. Therefore, a variety of hydrogenase mimics have been developed by researchers. However, to date, there are few ways to detect such hydrogenases. Simple, low-cost, and accurate analytical methods with high selectivity and sensitivity for the detection of hydrogenases need to be explored. Interestingly, CDs were found to form an inclusion complex with some of the hydrogenases [18,19,20,21].

Based on the above facts, herein, we aim to initially construct a strong emissive CD-based spatially confined AIE material by clicking the typically confined AIE molecule—tetraphenylethylene (TPE)—onto the β-CD primary face, investigating its fluorescence property, and applying it to hydrogenases sensing.

## 2. Materials and Methods

### 2.1. Materials

**Figure 1:**
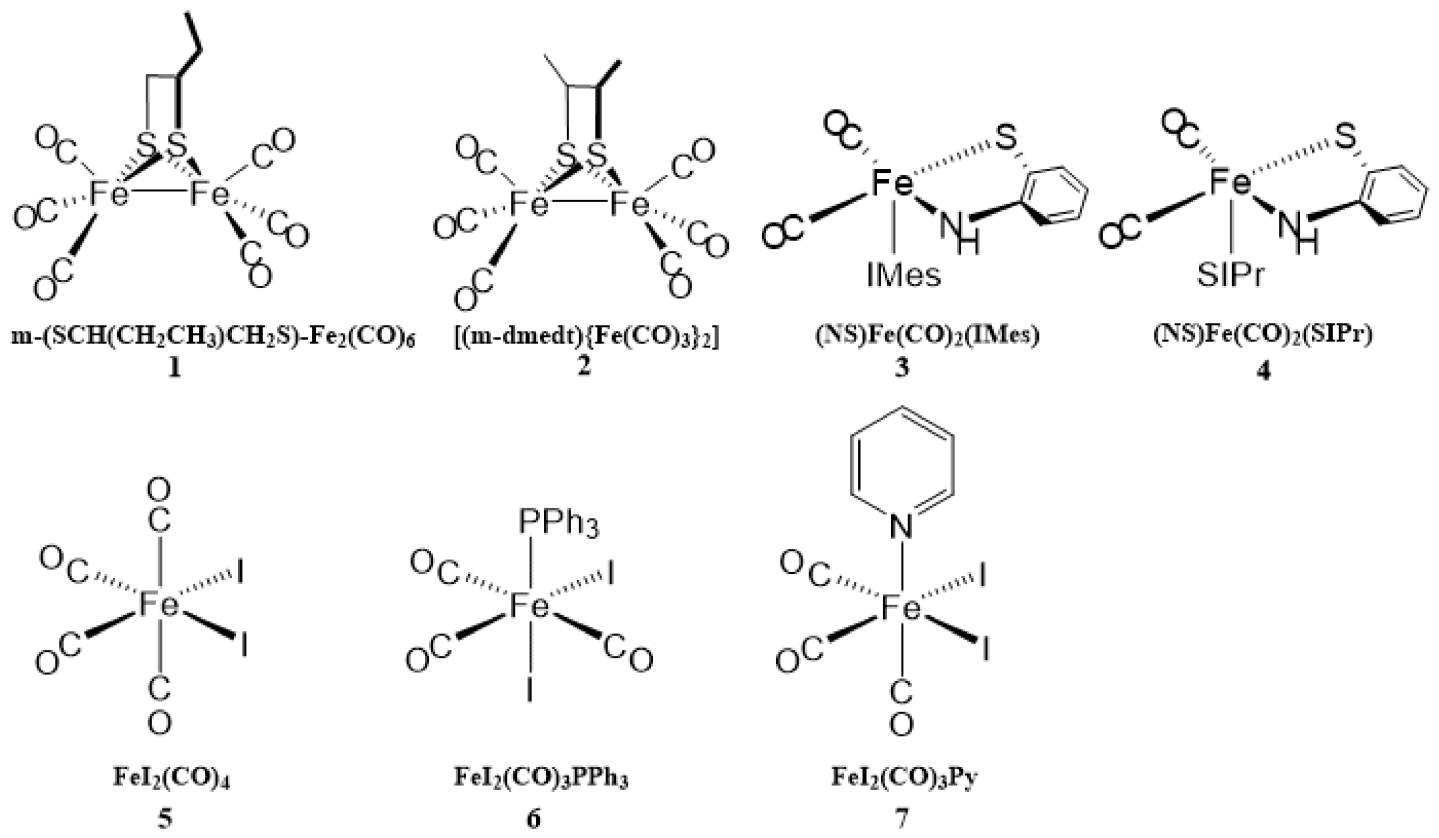
Chemical structures of hydrogenases **1**–**7**.

Diphenyl ketone, 4-hydroxy diphenyl ketone, propargyl bromide, tetrabutyl ammonium bromide, and sodium methoxide were purchased from HEOWNS (Tianjin, China). Sodium azide, CuSO_4_·5H_2_O, and pyridine were purchased from Tianjin Chemical Reagents (Tianjin, China). Tetrahydrofuran (THF), dimethyl sulfoxide (DMSO), methanol, and dichloromethane were purchased from Jiang Tian Chemical Reagents (Tianjin, China). Ascorbic acid sodium salt, iodine, and triphenylphosphine were provided by Energy-Chemical (Shanghai, China). THF was distilled over benzophenone and sodium prior to use. Other chemicals were of analytical grade and were used without further purification. The metal salts used for this study were CuCl_2_, FeCl_2_, NiCl_2_, ZnCl_2_, CoCl_2_, FeCl_3_, KCl, CaCl_2_, NaCl, MgCl_2_, AlCl_3_, Pb(NO_3_)_2_, HgCl_2_, BaCl_2_, and AgNO_3_. The anionic salts used for this study were NaF, NaCl, NaBr, NaNO_3_, Na_2_LiPO_4_, Na_2_CO_3_, Na_2_CrO_4_, Na_2_SO_3_, Na_2_SO_4_, Na_2_MoO_4_, NaH_2_PO_4_, NaHSO_3_, NaHSO_4_, and Na_2_S_2_O_3_. Deionized (DI) water was used in the experiments. The chemical structures of hydrogenases **1**–**7** are shown in Figure 1. According to the structures of the active sites, hydrogenases **1** and **2** were [FeFe]-hydrogenases, and hydrogenases **3**–**8** were [Fe]-hydrogenases. The hydrogenase analogues were synthesized and characterized according to our previous method [22,23,24,25] and stored below 10 °C.

### 2.2. Instrumentation

The ^1^H and ^13^C NMR were recorded on a Bruker ACF400 (400 MHz)—supplied by Bruker Biospin (Fällanden, Switzerland)—in deuterated chloroform (CDCl_3_), dimethylsulfoxide (DMSO-*d_6_*), and D_2_O—using tetramethylsilane (TMS) (*δ* = 0) as the internal reference. The Fouriertransform infrared (FTIR) spectra were collected on an AVATR360 supplied by Thermo Nicolet (Madison, WI, USA). The UV-VIS absorption spectra were obtained at room temperature using a UV-1800PC spectrophotometer. The Mass spectra were recorded on a LCQ Deca XP MAX system (Thermo Fisher, Waltham, MA, USA). The Elemental analysis was performed on a Var-io MICRO CHNOS elemental analyzer (Elementar Analysensysteme, Hanau, Germany). The fluorescence spectra were taken at room temperature on a Cary Eclipse fluorescence spectrophotometer supplied by Varian (Palo Alto, CA, USA).

### 2.3. Synthesis of the Spatially Confined AIECD (SCAIECD)

**Scheme 1:**
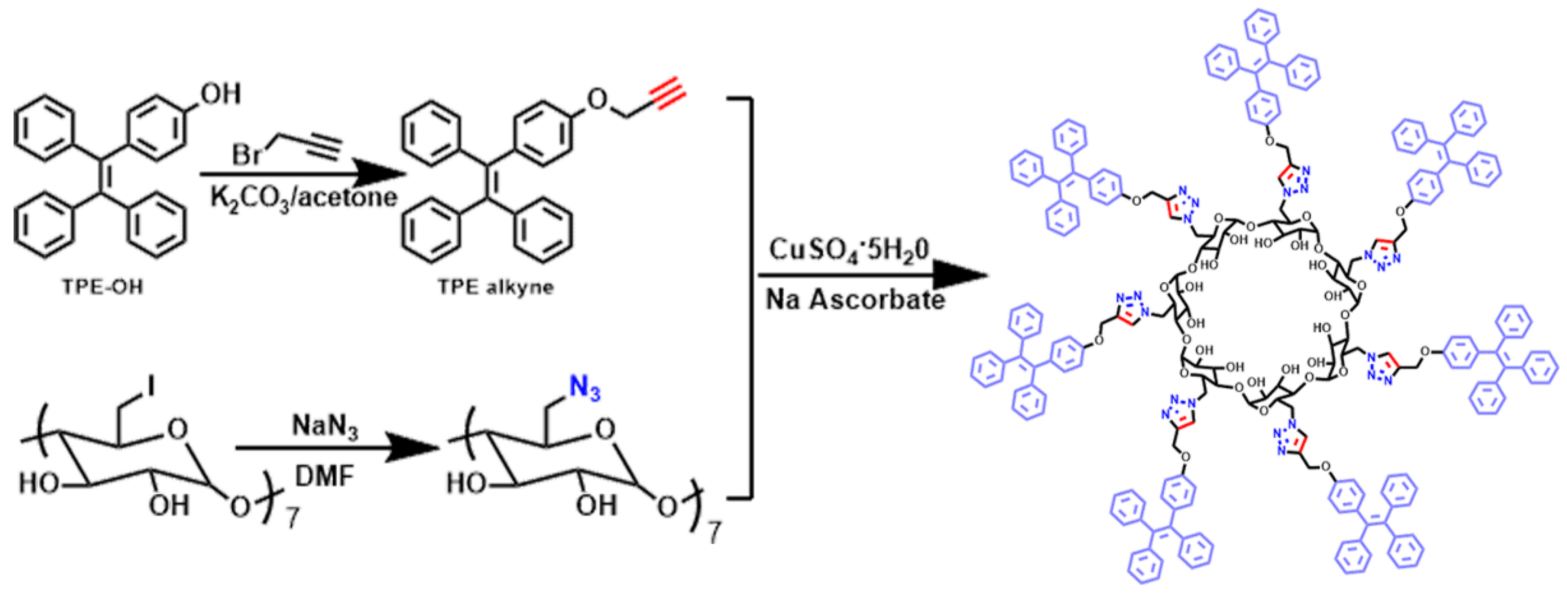
Synthetic pathway of the spatially confined aggregation induced emissioncy clodextrin (SCAIECD) (for detailed procedures, see the supporting information).

The synthetic pathways of the desired product are depicted in Scheme 1. TPE-alkyne and heptakis(6-deoxy-6-azido)-β-CD were used as the click precursors. The 1-(4-hydroxyphenyl)-1,2,2-triphenylethylene—(TPE-OH) synthesized via the McMurry coupling reaction—reacted with propargyl bromide in acetone in the presence of K_2_CO_3_ to afford TPE-alkyne. Heptakis(6-deoxy-6-azido)-β-CD was obtained by reacting heptakis(6-deoxy-6-iodo)-β-CD with sodium azide [26,27], with a yield of 95%. The novel SCAIECD, with the confinement effect, was finally prepared via click chemistry [28,29,30] between TPE-alkyne and heptakis(6-deoxy-6-azido)-β-CD, with a yield of 90%. Detailed synthetic procedures and characterization data (Figures S1–S8) are included in the supporting information. Furthermore, a single TPE-substituted CD molecule without spatial confinement (AIECD) was synthesized for comparison in this study (for synthetic characterization data, see the supporting information).

## 3. Results and Discussion

### 3.1. Structure Characterization and AIE Property Study of SCAIECD and AIECD

The correct structures of SCAIECD and AIECD were confirmed by FTIR and NMR. As shown in Figure S7a, the FTIR of heptakis(6-deoxy-6-azido)-β-CD indicated a very strong typical signal, around 2110 cm^−1^. After the click reaction, this AIECD signal still exhibited strong absorption (Figure S7b), while it disappeared in the FTIR of SCAIECD (Figure S7c), which indicated that all of the moieties had been converted to triazoles. ^1^H NMR (Figures S8 and S9) showed that SCAIECD and AIECD all exhibited typical TPE proton signals (around 7 ppm), revealing the successful immobilization of TPE onto the CD skeletons. The elemental analysis results indicated that the C%, H%, and N% were 73.14% (73.28%, calculated), 5.52% (5.45%, calculated), and 7.40% (7.33%, calculated), respectively. As the synthesized CD derivatives had good solubility in DMSO but were not dissolvable in water, we chose an H_2_O/DMSO solvent system to investigate their AIE feature initially, while AIECD was chosen for comparison.

**Figure 2:**
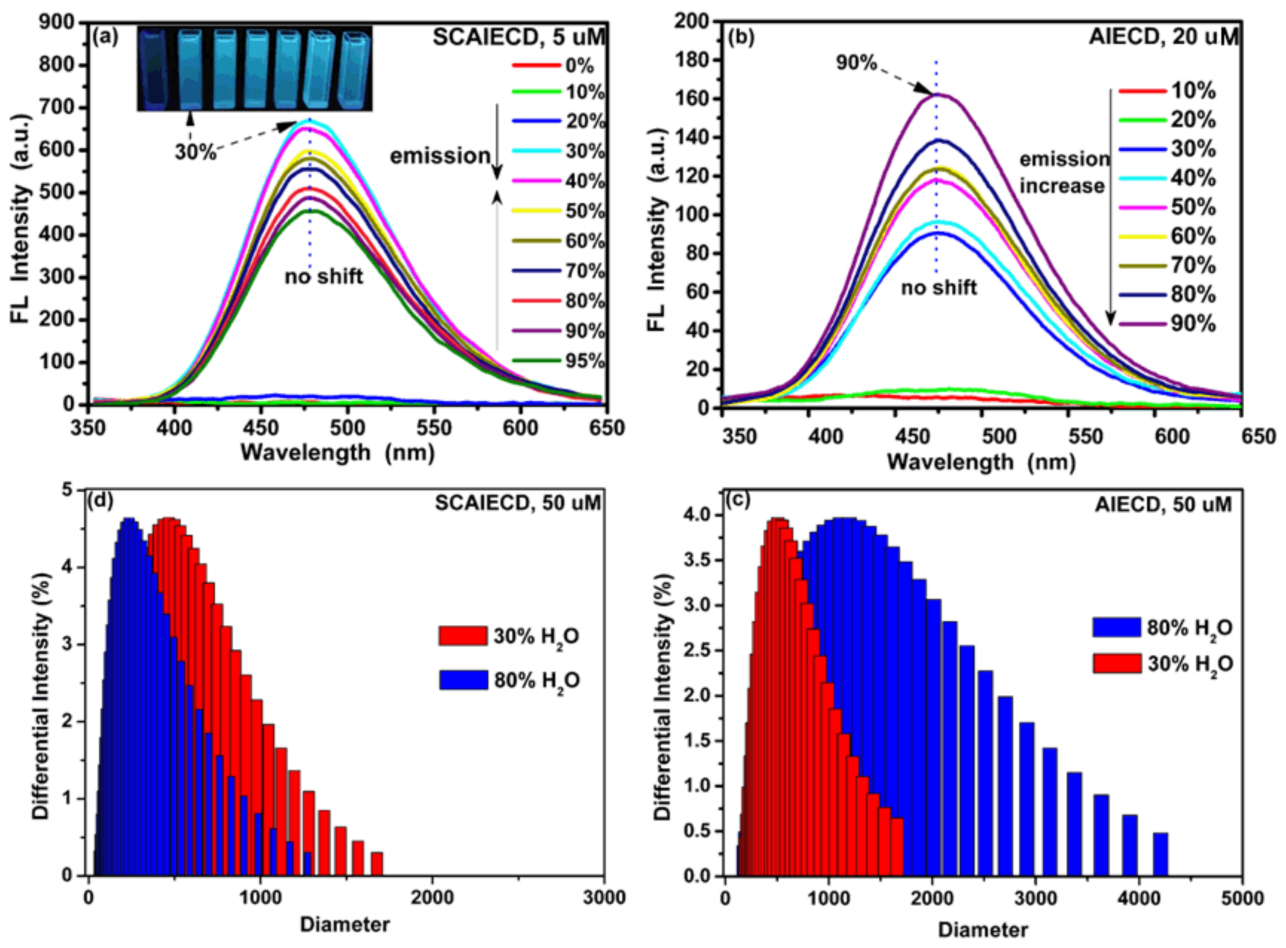
(**a**) Fluorescence emission spectra for solutions of SCAIECD (5 µM) in different ratios of DMSO/H_2_O (*v*/*v*); (**b**) fluorescence emission spectra for solutions of AIECD (20 µM) in different ratios of DMSO/H_2_O (*v*/*v*); (**c**) DLS results of SCAIECD (5 µM) in DMSO/H_2_O (*v*/*v*); and (**d**) DLS results of AIECD (50 µM) in DMSO/H_2_O (*v*/*v*).

As shown in Figure 2a, when excited at 330 nm, SCAIECD did not emit any fluorescence in its DMSO solution and the FL intensity showed no change with the increase of water fraction from 0 to 20% (DMSO/H_2_O, *v*/*v*). However, a critical point appeared in the DMSO/H_2_O solution (70/30, *v*/*v*), where a rapid emission increase took place and the FL intensity almost reached 700 a.u.—with 30% water content and a low concentration of 5 µM. Surprisingly, the FL intensity gradually decreased with the further increase of the water fraction from 30% to 95% (DMSO/H_2_O, *v*/*v*). While for AIECD, a regular AIE characteristic was found, where its emission was gradually enhanced with the increase of water content from 0% to 90% (DMSO/H_2_O, *v*/*v*) and where the maximum FL intensity was only 160 a.u. with a high concentration of 20 µM. The unique AIE characteristic of SCAIECD was undoubtedly ascribed to the spatial confinement effect. On one hand, the pre-aggregated state of TPE on the CD primary face significantly enhanced the water sensitivity of SCAIECD in a DMSO/H_2_O solution (70/30, *v*/*v*), indicating that a stronger solvent was sufficient to turn on its florescence emission, which had never been reported anywhere; on the other hand, the spatial confinement might have resulted in a tighter aggregation state of TPE luminogens [31,32], thus remarkably restricting the intramolecular rotation of the TPE phenyl rings, leading to the extraordinary strong emission at low concentrations. As expected, AIECD showed a particle size increase with increasing water fraction from 30% to 80% (DMSO/H_2_O, *v*/*v*) accompanied with the florescence enhancement (Figure 2c). However, the slight emission decrease after the emission maxima of SCAIECD might be because of the reduced particle size caused by the hydrogen bond between CD and H_2_O molecules, which was verified by the comparable DLS results (Figure 2d). Furthermore, unlike typical AIE luminogens, it was interesting to find that the emission of SCAIECD and AIECD did not show any bathochromic-shift phenomenon during the gradual increase of a poor solvent, revealing that CD satisfied the rigid restrictions to fix the AIE luminogen’s emission wavelength, which was favorable for sensing applications.

### 3.2. Evaluation of the Sensing Ability of SCAIECD for Hydrogenases

According to the above experimental results, the sensing ability of SCAIECD for hydrogenases was evaluated using H_2_O/DMSO at the concentration of 5 µM. TPEs acted as the fluorescent unit and the CD cavity acted as the host for the inclusion of hydrogenases.

**Figure 3:**
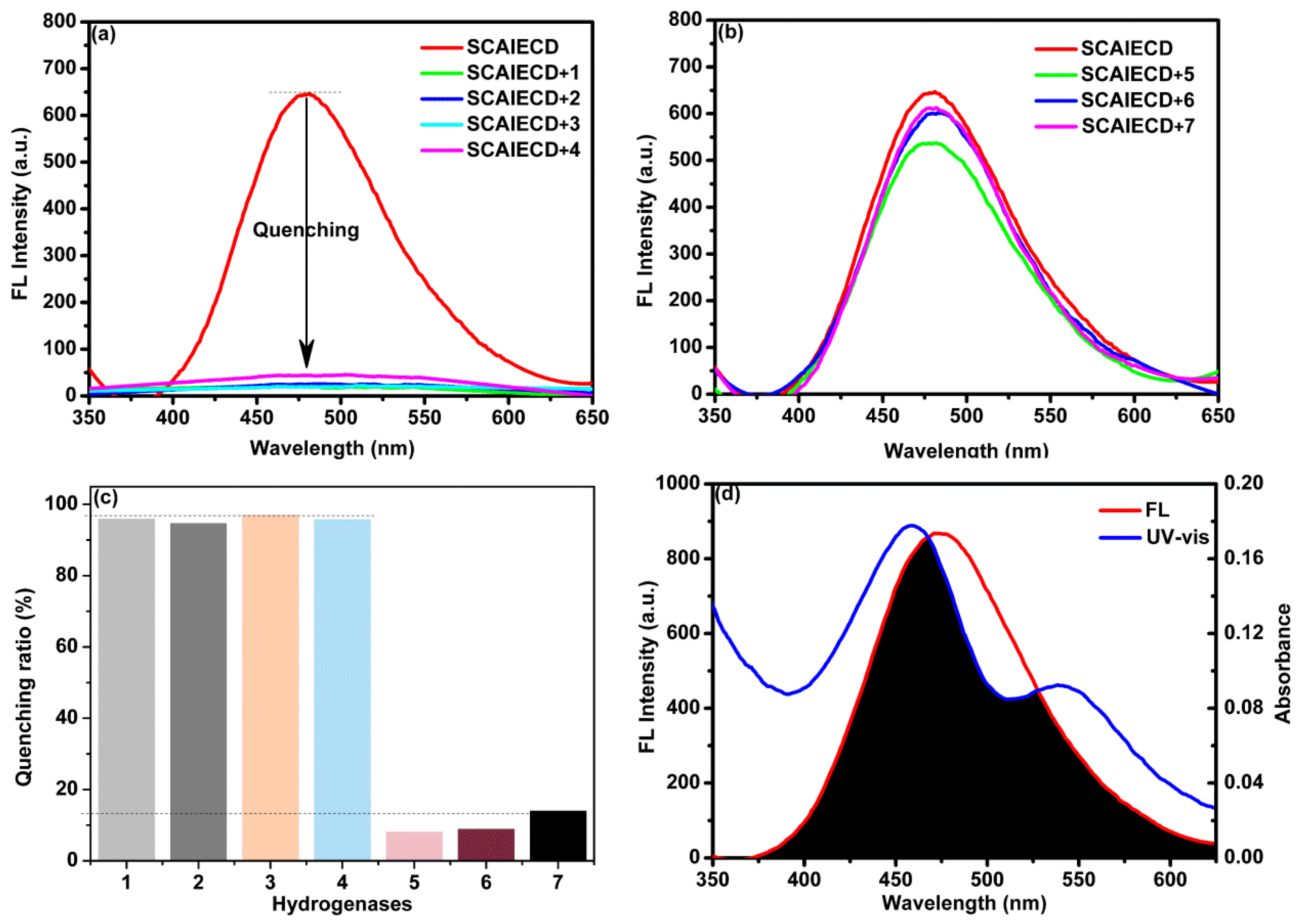
(**a**,**b**) Fluorescence emission spectra upon addition of hydrogenases (5 µM) to a solution of **SCAIECD** (5 µM); (**c**) Fluorescence quenching ratio upon addition of hydrogenases (5 µM) to a solution of **SCAIECD** (5 µM); and (**d**) fluorescence emission spectra of **SCAIECD** in DMSO/H_2_O (70/30, *v*/*v*) and the UV-VIS of hydrogenase **1**.

Interestingly, it was observed that the strong fluorescence of SCAIECD was significantly decreased when it was exposed to hydrogenases solutions, which revealed the great potential for SCAIECD to detect hydrogenases. By altering the DMSO content, we found that SCAIECD was most sensitive to hydrogenases in a DMSO/H_2_O solution (70/30, *v*/*v*) where SCAIECD exhibited emission maxima. To investigate the versatility of the novel sensor, we studied the quenching ability of different hydrogenases, and the results are depicted in Figure 3a–c. Interestingly, as shown, the fluorescence intensity of SCAIECD was significantly quenched; when one molar equivalent of hydrogenases **1**, **2**, **3**, or **4** was introduced into the solution, the quenching ratios were over 90%. However, the fluorescence intensity was only slightly reduced when it was introduced to hydrogenases **5**, **6**, or **7**, resulting in small quenching ratios (below 10%). Figure 3d shows the UV-VIS absorption bands of hydrogenase **1** and the fluorescence spectrum of SCAIECD. We found that a strong overlap existed between the absorption and emission peak. As is known, the CDs showed an exceptional complexation ability with some transition metal derivatives to form inclusion complexes. By closely looking at the structures of hydrogenases, it was interesting to find that **1**, **2**, **3**, and **4** all have Fe–S bonds in their skeletons when compared with **5**, **6**, and **7**.

**Figure 4:**
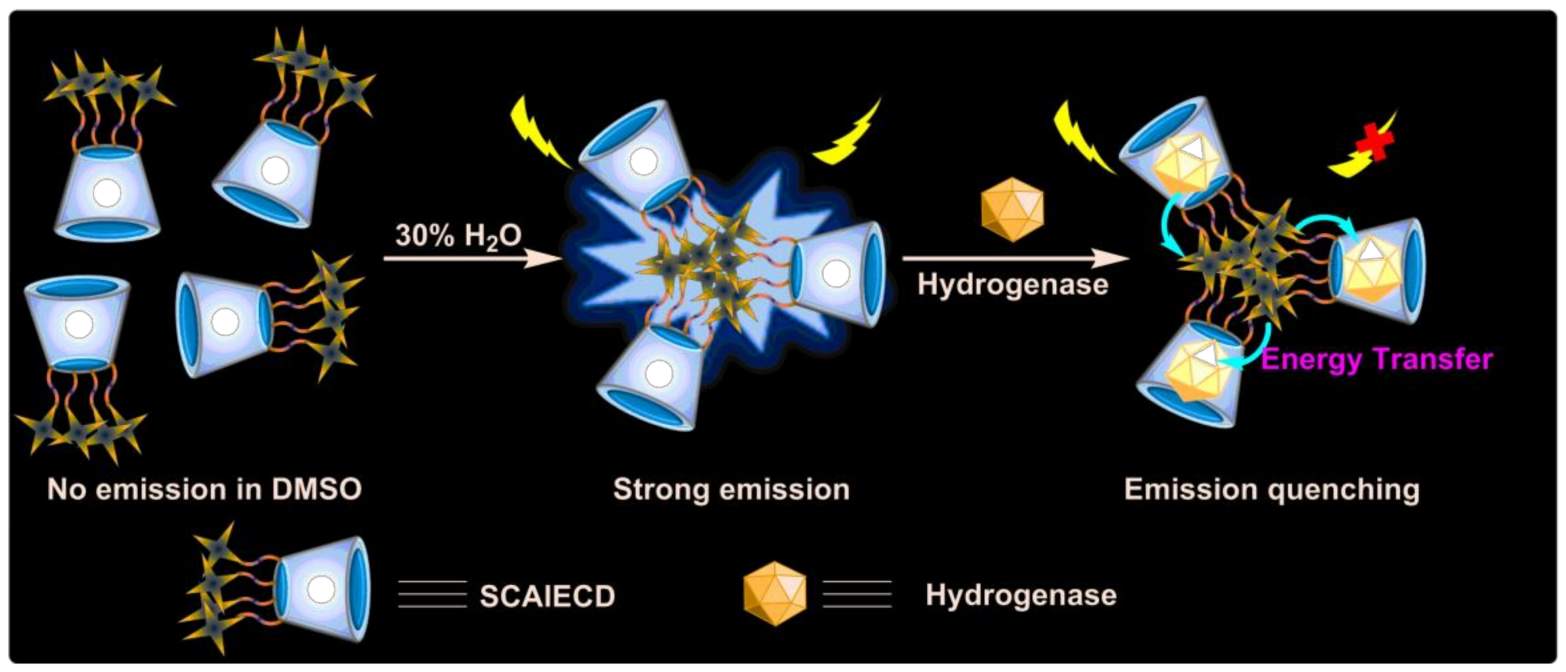
Sensing mechanism of **SCAIECD**.

According to the above experiments, the florescence quenching mechanism (Figure 4) of SCAIECD might have been similar to the fluorescence resonance energy transfer (FRET), where the energy was transferred from the aggregated TPE molecule’s excited state to the Fe–S contained hydrogenases—which are close enough to the CD primary face caused by the inclusion formation [33]. This interesting phenomenon could be used to produce a photo-switchable supermolecular CD system which will be further studied. The above results indicated that the confined AIE cyclodextrin could serve as good sensors for selective detection of hydrogenases.

### 3.3. Evaluation of the Detection Limit and Reliability

**Figure 5:**
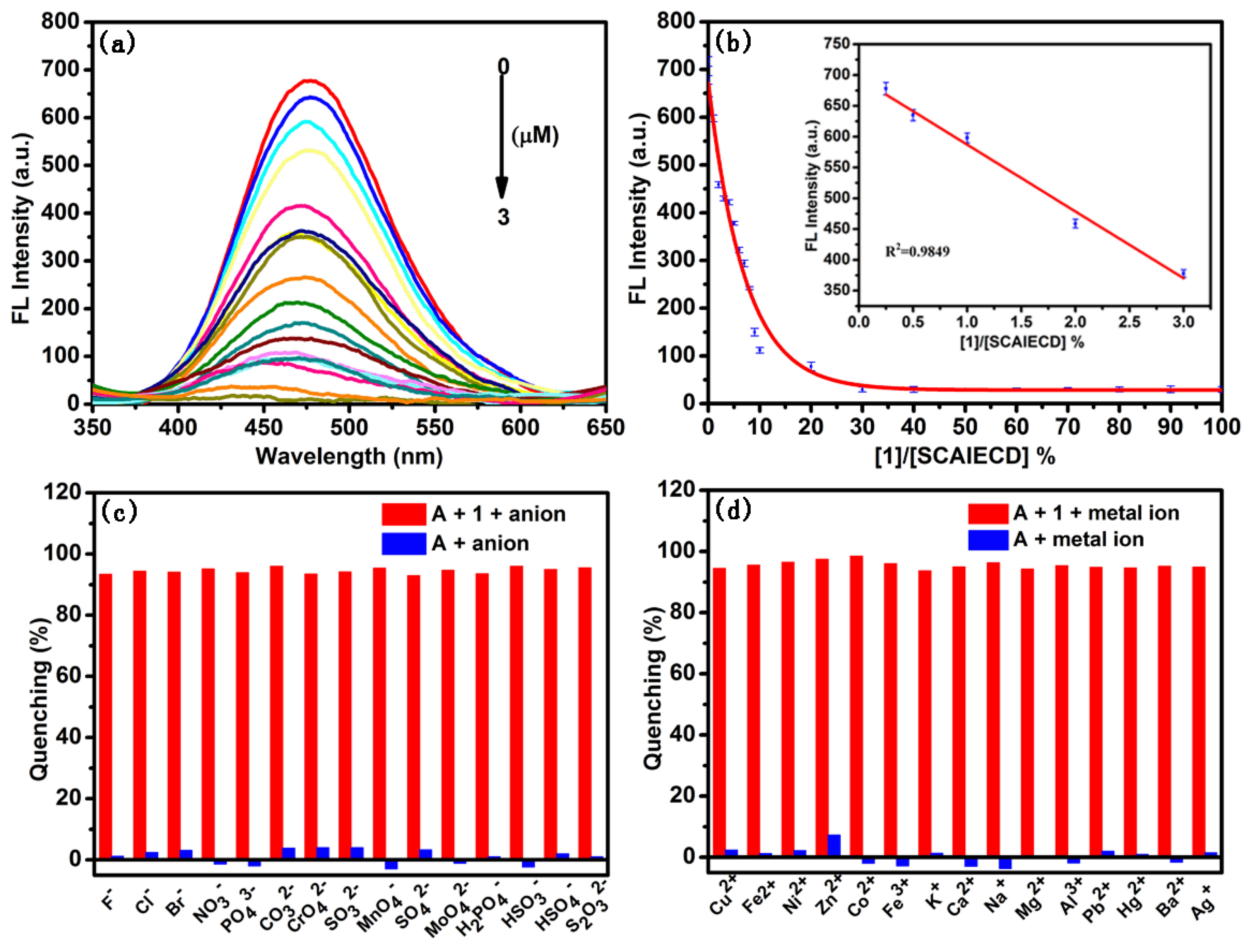
(**a**) Fluorescence emission spectra of SCAIECD (5 µM) exposed to various concentrations of 1 (0, 0.001, 0.01, 0.05, 0.1, 0.2, 0.3, 0.4, 0.5, 0.6, 0.7, 0.8, 0.9, 1, 1.5, 2, 2.5, and 3 µM from top to bottom); (**b**) fluorescence titration curve of SCAIECD with the hydrogenase of **1**, the inset shows the fluorescence response at low hydrogenase concentrations; (**c**) percentage fluorescence quenching of SCAIECD (5 µM) with different metal ions and the hydrogenase of 1 (red bars), percentage fluorescence quenching of SCAIECD (5 µM) only in the presence of different metal ions (blue bars); (**d**) percentage fluorescence quenching of SCAIECD (5 µM) with different anions and the hydrogenase of **1** (red bars), percentage fluorescence quenching of SCAIECD (5 µM) only in the presence of different anions (blue bars). Note: DMSO/H_2_O, 70/30, *v*/*v*; λex = 330 nm, ex/em slits = 5/5. A represents SCAIECD.

In order to figure out the detection limit, fluorescence titration was conducted in the current study using hydrogenase 1 as the model analyte. As shown (Figure 5a,b), with the concentration increase of 1, the fluorescence intensity of SCAIECD was gradually quenched and the fluorescence was completely diminished when the hydrogenase concentration increased to 0.6 molar equivalent of SCAIECD’s concentration. According to our previous research [34], when we conducted the titration experiment, a good linear response was found in the low concentration range (Figure 5b inset) and the detection limit could be to 3 µM.

The interference of various ions in the current assay were also investigated. Figure 5c depicts the fluorescence quenching ratio of the sensor in the presence of anions. Figure 5d depicts the fluorescence quenching ratio of the sensor in the presence of metal ions. As can be seen, the changes in the fluorescence quenching ratio of SCAIECD were not significant in the presence of the studied anions and metal ions, indicating that these ions had negligible interfering effects on the sensing process for SCAIECD. To further investigate the selectivity of the SCAIECD towards hydrogenases, the effect of some substances which could potentially enter the cavity of cyclodextrin was evaluated, including l-Phenylalanine, d-Phenylalanine, 2-Aminophenol, 1-naphthalenol, 4-aminobenzoic acid, l-Tryptophane, and P-phthalic acid. As shown in Figure S12, all of the studied substances had little effect on the detection of hydrogenase. The results revealed that SCAIECD showed good selectivity and reliability for **1**, **2**, **3**, and **4**, and would be satisfactorily used to detect them.

## 4. Conclusions

In summary, a spatially confined AIE material has been successfully prepared by clicking TPE molecules to the primary face of β-CD. Spatial confinement is a good alternative to prepare highly emissive AIE compounds. The built supramolecular AIECD derivative provides a satisfactory supramolecular platform for the selective detection of hydrogenases via the inclusion complexation and energy transfer from the AIE emission moieties to the included hydrogenase guests. Although the exact quenching mechanism still needs further investigation, we believe that SCAIECD has the potential for applications in biochemistry and medicinal chemistry, due to its possible biocompatibility and ability to accommodate a large number of guest molecules.

## Supplementary Materials

The following are available online at http://www.mdpi.com/1424-8220/18/4/1134/s1.
