# Supplementary material for: Click Access to a Cyclodextrin-Based Spatially Confined AIE Material for Hydrogenase Recognition
Source: Sensors (Basel). 2018 Apr 8;18(4):1134. doi: 10.3390/s18041134 (PMC5948543; doi:10.3390/s18041134)
Supplement: Supplementary file 1 [file sensors-18-01134-s001.pdf]

SupplementaryMaterials: Click access to a cyclodextrin-based spatially confined AIE material for hydrogenase recognition

Rui Zhao, Bin Li, Yong Wang* and Wenping Hu

Synthesis and Characterization S1–S12

1. Synthesis of 1-(4-hydroxyphenyl)-1,2,2-triphenylethylene

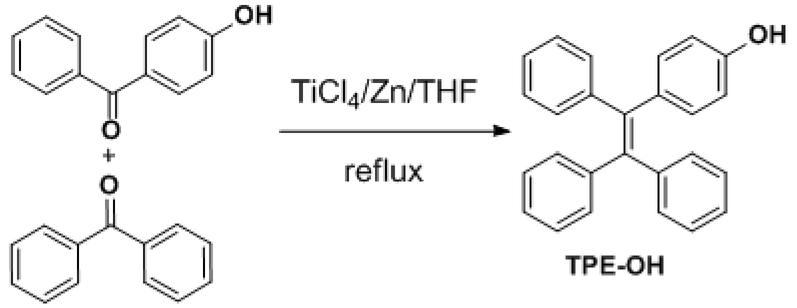

Under an nitrogen atmosphere, a three-necked flask equipped with a magnetic stirrer was charged with zinc powder (1.6 g, 24 mmol) and 40 mL dry THF. The mixture was cooled −5 to 0℃, and $TiCl_4$ (1.3 mL, 12mmol) was slowly added by a syringe with the temperature kept under 10℃. The suspending mixture was warmed to room temperature and stirred for 0.5 h, then heated at reflux for 2.5 h. The mixture was again cooled to −5 to 0℃, charged with pyridine (0.5 mL, 6 mmol) and stirred for 10 min. The solution of diphenyl ketone and 4-hydroxy diphenyl ketone [in 1:1.2 mole ratio, 2.4 mmol] in 15 mL THF was added slowly. After addition, the reaction mixture was heated at reflux until the carbonyl compounds were consumed (monitored by TLC). The reaction was quenched with 10% K_2CO_3 aqueous solution and taken up with CH_2Cl_2. The organic layer was collected. After solvent evaporation, the crude product was purified on a silica gel column using ethyl acetate/petroleum ether (1:10, *v*/*v*) as eluent to give the desired product as a white powder in a yield of 76%. ^{1}HNMR (400 MHz, $CDCl_3$): δ (ppm) 4.82 (s, 1H), 6.56 (d, 2H), 6.90 (d, 2H), 6.98-7.16 (m, 15H). ^{13}CNMR (400 MHz, $CDCl_3$): δ (ppm) 1114.63, 126.42, 126.60, 127.65, 127.75, 131.36, 131.38, 131.40, 132.76, 136.37, 140.21, 140.48, 143.93, 143.94, 144.04, 154.08; ESI (+)-MS: calcd. for $C_{26}H_{20}O$: 347 [M]; found 348.9 [M+H]+; IR (ν, KBr): 1246, 1441, 1516 and 3345 cm^{-1}.

2. Synthesis of TPE-alkyne

The mixture of compound 1 (0.45 g, 1.3 mmol), propargyl bromide (0,225 mL, 1.95 mmol), K_2CO_3 (0.699 g, 5.07 mmol) and NBu_4Br (3.2 mg, 0.001 mmol) in acetone (15 mL) was refluxed overnight under nitrogen. The mixture was then filtered and dried over anhydrous $MgSO_4$. After the solvent evaporation, the crude product was purified by a silica gel column using ethyl acetate/petroleum ether (1:15, *v*/*v*) as eluent affording a light yellow syrup in a yield of 95%. ^{1}HNMR (400MHz, $CDCl_3$): δ(ppm) 2.52 (s, 2H), 4.84 (d, 4H), 6.72 (m, 2H), 6.96 (m, 2H), 7.14-7.03 (m, 15H). ^{13}CNMR (400 MHz, $CDCl_3$): δ(ppm) 55.8, 75.4, 78.6, 113.6, 126.5, 127.6, 131.3, 132.5, 137.1, 140.6, 156.3.. ESI (+)-MS: calcd. for $C_{29}H_{22}O$: 386 [M]; found 387.5 [M+H]+.

3. Synthesis of Heptakis(6-deoxy-6-iodo)-β-CD

PPh_3 (11.5 g, 44 mmol) was dissolved in dry DMF (60 mL) in a 250 mL flask equipped with a condenser under N_2. During the heating at 50°C, I_2 was added carefully (11.2 g, 44 mmol). β-cyclodextrin (2.5 g, 2.2 mmol) was then added to the dark brown solution and the solution was stirred at 85°C for 18h under N_2. After the reaction, the solution was concentrated under reduced pressure by the removal of DMF. The reaction mixture was then cooled to room temperature. Then the reaction flask was cooled with an ice-bath and a solution of sodium methanoate (2.8 g in 40 mL)

was added to the mixture. The mixture was stirred few minutes, then precipitated in 600 mL of methanol. The insoluble yellow product was filtered and washed with methanol, until the solvent becomes colorless. The product was dried at 80°C under vacuum overnight with a yield of 90%. ^{1}H NMR (DMSO-d_6): δ (ppm): 6.03 (d, 14H), 5.94 (s, 7H), 4.99 (d, 7H), 3.82-3.27 (m, 42H).

4. Synthesis of Heptakis(6-deoxy-6-azido)-β-CD

Sodium azide (1.4 g, 21 mmol) was added to a solution of heptakis(6-deoxy-6-iodo)- -cyclodextrin (2.0 g, 1.05 mmol) in dry DMF (90 mL), in a 250 mL flask equipped with a condenser under N_2. The solution was stirred at 90 ℃ for 24 h. The solution was then concentrated under reduced pressure. The mixture was precipitated in 500 mL of water. The white insoluble product was filtered and washed with large excess of water. The product was dried at 80℃ under vacuum overnight with a yield of 95%. ^{1}H NMR (DMSO-d_6): δ (ppm): 6.03 (14H), 5.9 (7H), 4.9 (7H), 3.8-3.2 (42H). ^{13}C NMR (400 MHz, $CDCl_3$): δ (ppm) 51.81, 70.79, 72.48, 73.05, 83.67, 102.53. ESI(+)-MS: calcd.for $C_{42}H_{63}O_{28}N_{21}$: 1132.4 [M]; found 1132.4 $[M+H]^+$. IR (ν, KBr): 3394.22, 2926.22, 2108.19, and 1047.83 cm^{-1}

5. Synthesis of SCAIECD via Click Chemistry

Heptakis(6-deoxy-6-azido)-β-CD (0.4 g, 0.3 mmol) was added to a solution of TPE-alkynl (1.64 g, 4.2 mmol) in DMF (55 mL) followed by addition of the aqueous solution of $CuSO_4{\cdot}5H_2O$ and ascorbic acid sodium salt. The reaction mixture was stirred at 90℃ for 2 days under nitrogen atmosphere. After removing the solvent, the crude product was washed by water and dried over $MgSO_4$. After the solvent evaporation, the residue was then purified over silica gel chromatography (without pressure) using ethyl acetate/petroleum ether (1:10, *v/v*) (to remove the excess of TPE-alkynl) followed by a mixture of water and methanol (1:2, *v/v*). Brown solid was obtained in a yield of 80%.^{1}H NMR (400 MHz, DMSO-d_6) δ (ppm): 8.38 (s, 7H), 8.02 (s, 7H), 7.98 (s, 7H), 6.95-7.14 (m, 119H), 6.78-6.80 (d, 14H), 6.55-6.57 (d, 14H), 6.01-6.08 (d, 14H), 5.14 (s, 7H), 4.95 (s, 7H), 4.67 (d, 7H), 4.08-4.30 (m, 7H), 3.42-3.75 (m, 28H). IR (ν, KBr): 3387.16, 2961.29, 1604.93, 1503.25 and 1041.89, 806.06, 696.21 cm^{-1}.

6. Synthesis of AIECD Via Click Chemistry

Heptakis(6-deoxy-6-azido)- -CD (0.4 g, 0.3 mmol) was added to a solution of TPE-alkynl (0.117 g, 0.3 mmol) in DMF (55 mL) followed by addition of the aqueous solution of $CuSO_4{\cdot}5H_2O$ and ascorbic acid sodium salt. The reaction mixture was stirred at 90℃ for 2 days under nitrogen atmosphere. After removing the solvent, the crude product was washed by water and dried over $MgSO_4$. After the solvent evaporation, the residue was then purified over silica gel chromatography (without pressure) using ethyl acetate/petroleum ether (1:10, *v/v*) (to remove the excess of TPE-alkynl) followed by a mixture of water and methanol (1:2, *v/v*). Brown solid was obtained in a yield of 80%.^{1}H NMR (400 MHz, DMSO-d_6) δ (ppm): 6.75-7.09 (m, 15H), 5.90-5.91 (m, 14H), 4.92 (s, 7H), 3.34–3.79 (m, 42H). IR (ν, KBr): 3351.10, 2923.32, 2107.61, 1504.51 and 1045.17, 804.10, 699.22 cm^{-1}.

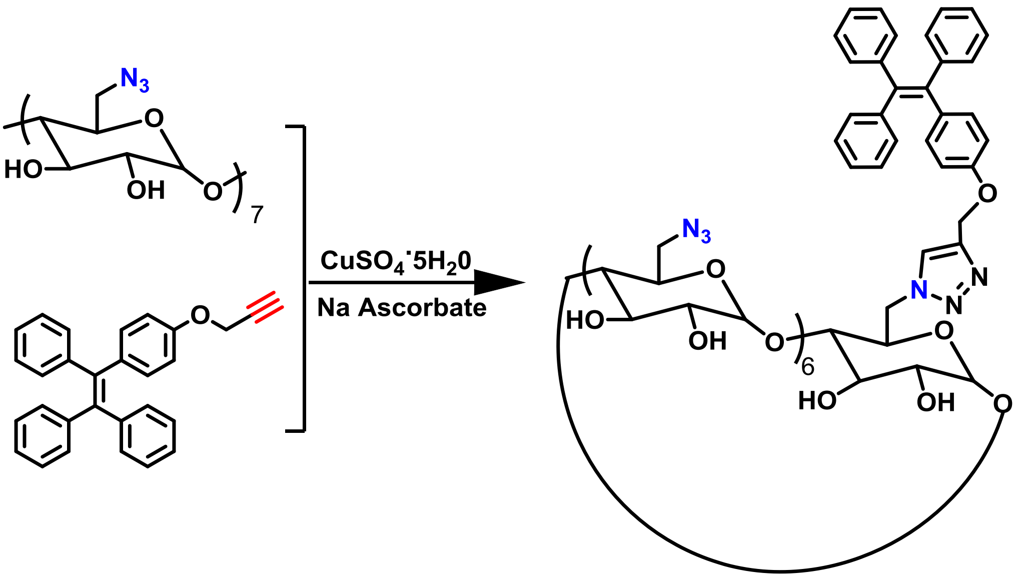

Schematic. Synthetic pathway of the AIECD.

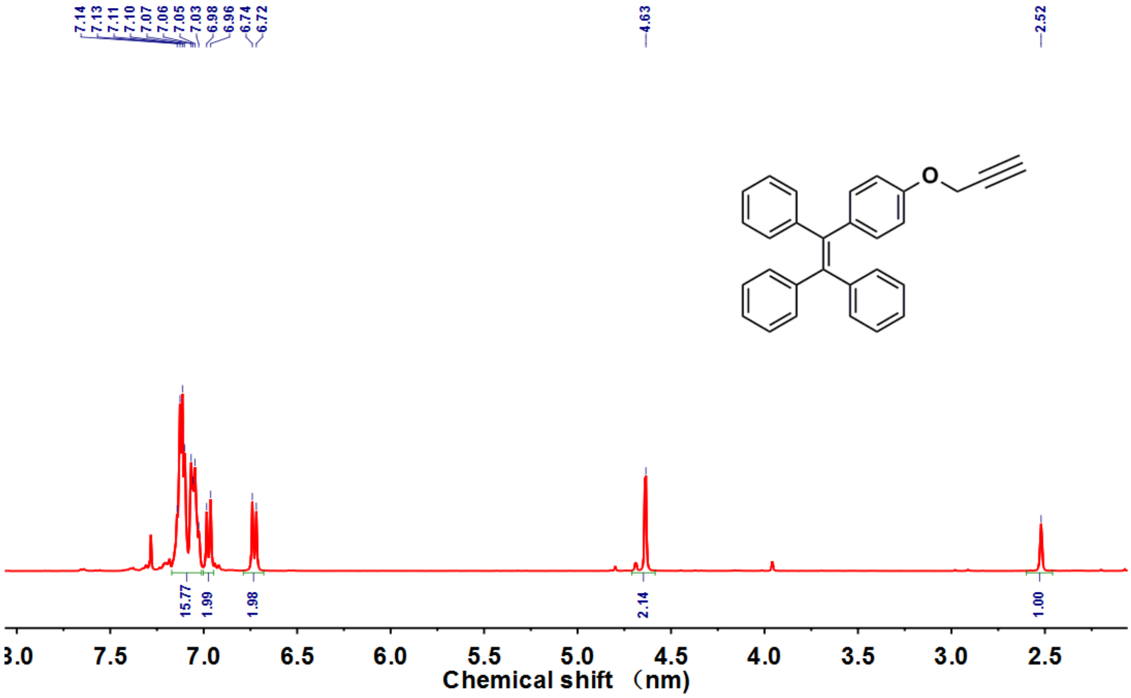

Figure S1. ^{1}H NMR of TPE-alkyne in $CDCl_3$.

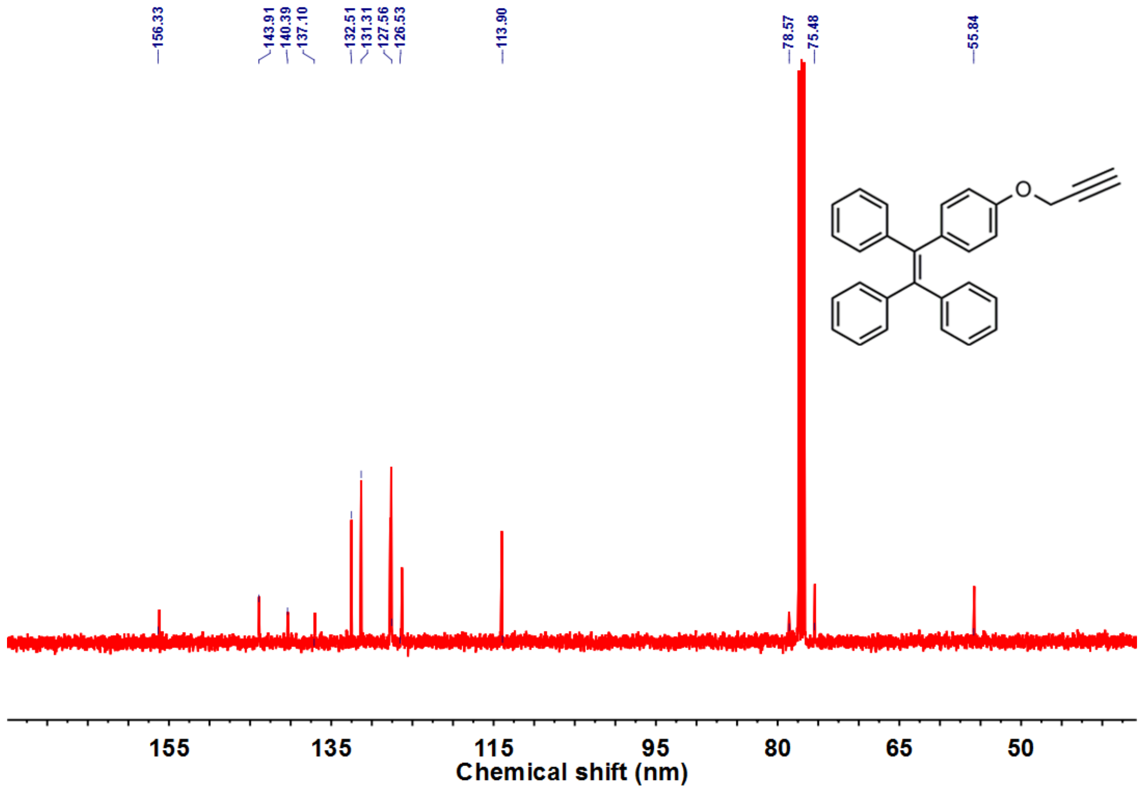

Figure S2.^{13}C NMR of TPE-alkyne in $CDCl_3$.

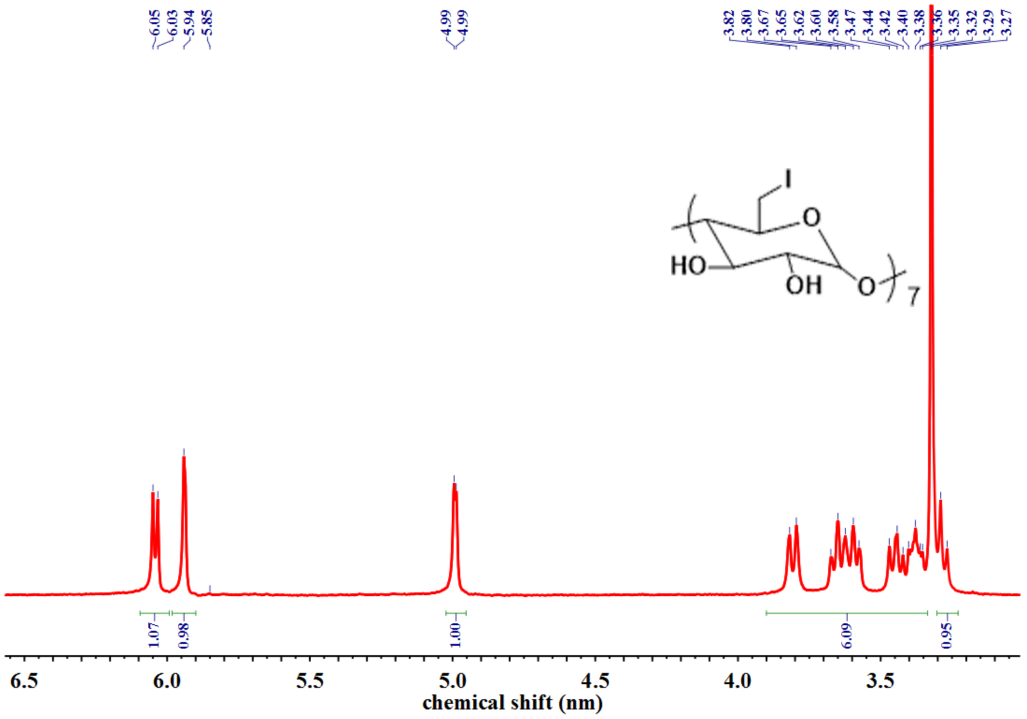

Figure S3.^{1}H NMR of heptakis(6-deoxy-6-iodo)-β-CD in DMSO-d_6.

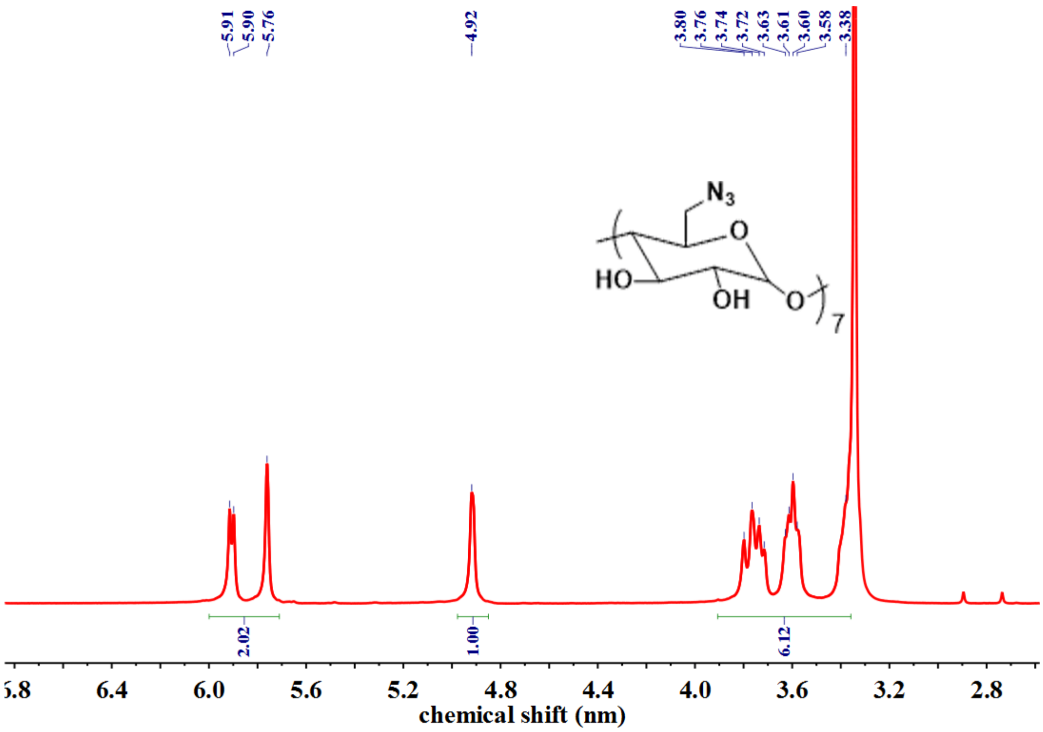

Figure S4.^{1}H NMR of heptakis(6-deoxy-6-azido)-β-CD in DMSO-d_6.

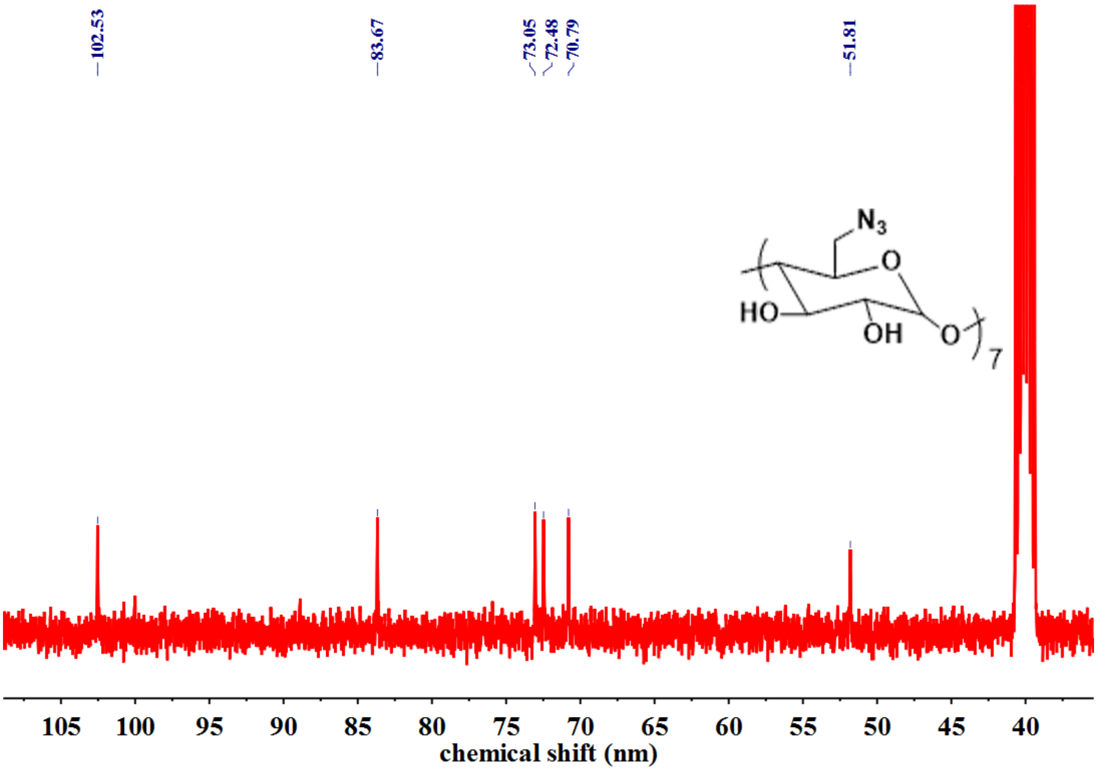

Figure S5.^{13}C NMR of heptakis(6-deoxy-6-azido)-β-CD in DMSO-d_6.

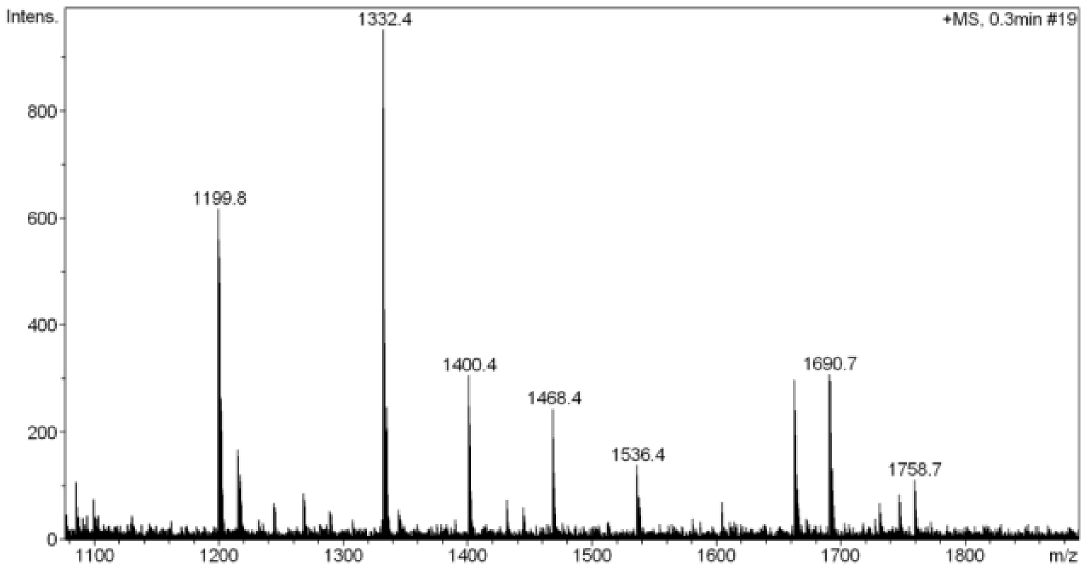

Figure S6. Mass spectra of haptakis(6-deoxy-6-azido)-β-CD.

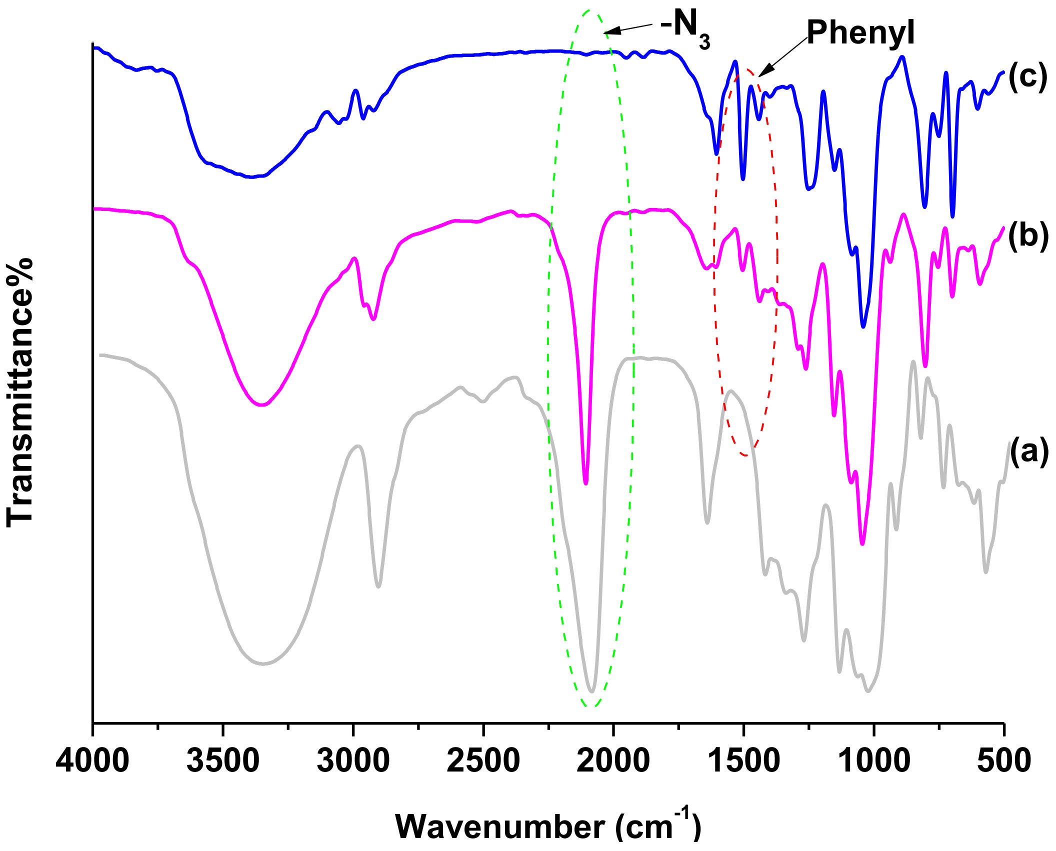

Figure S7.IR spectrum of heptakis(6-deoxy-6-azido)-β-CD (**a**); SCAIECD (**b**); AIECD (**c**).

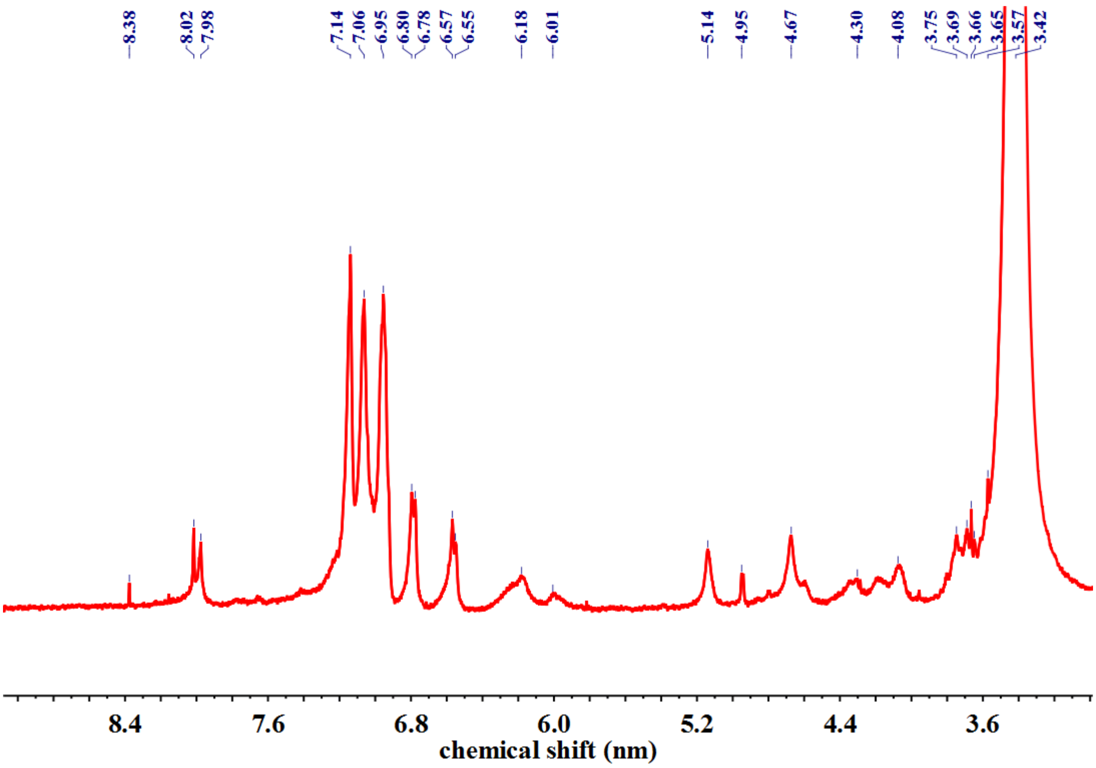

Figure S8.¹H NMR of**SCAIECD** in DMSO-*d*6.

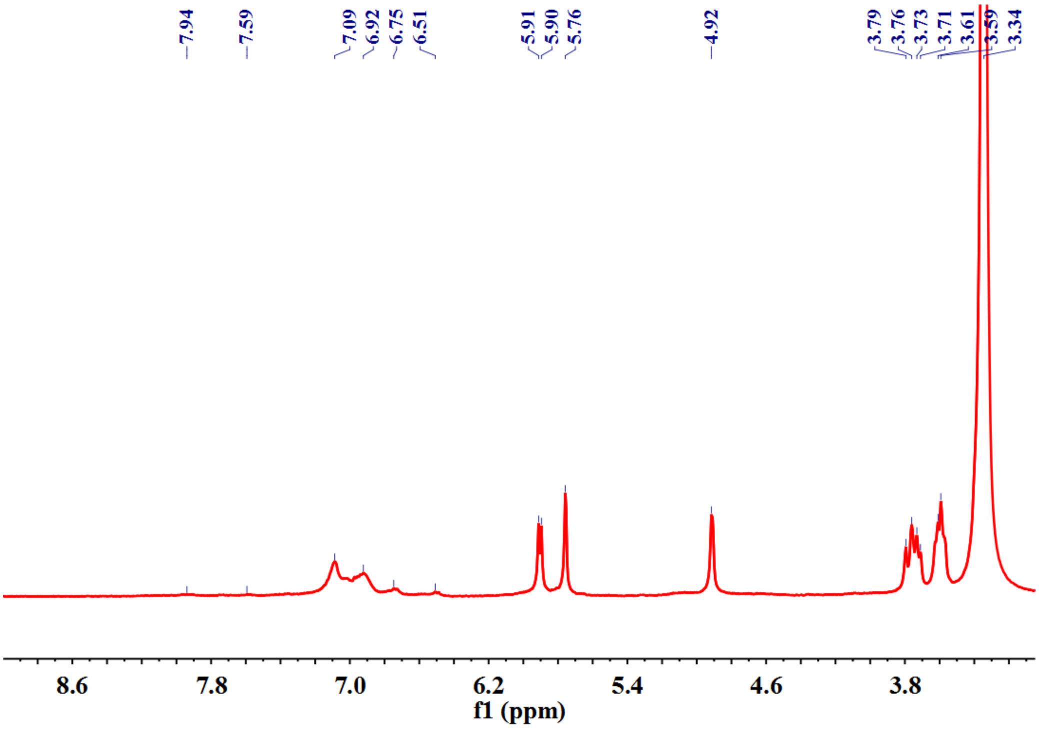

Figure S9.¹H NMR of**AIECD** in DMSO-*d*6.

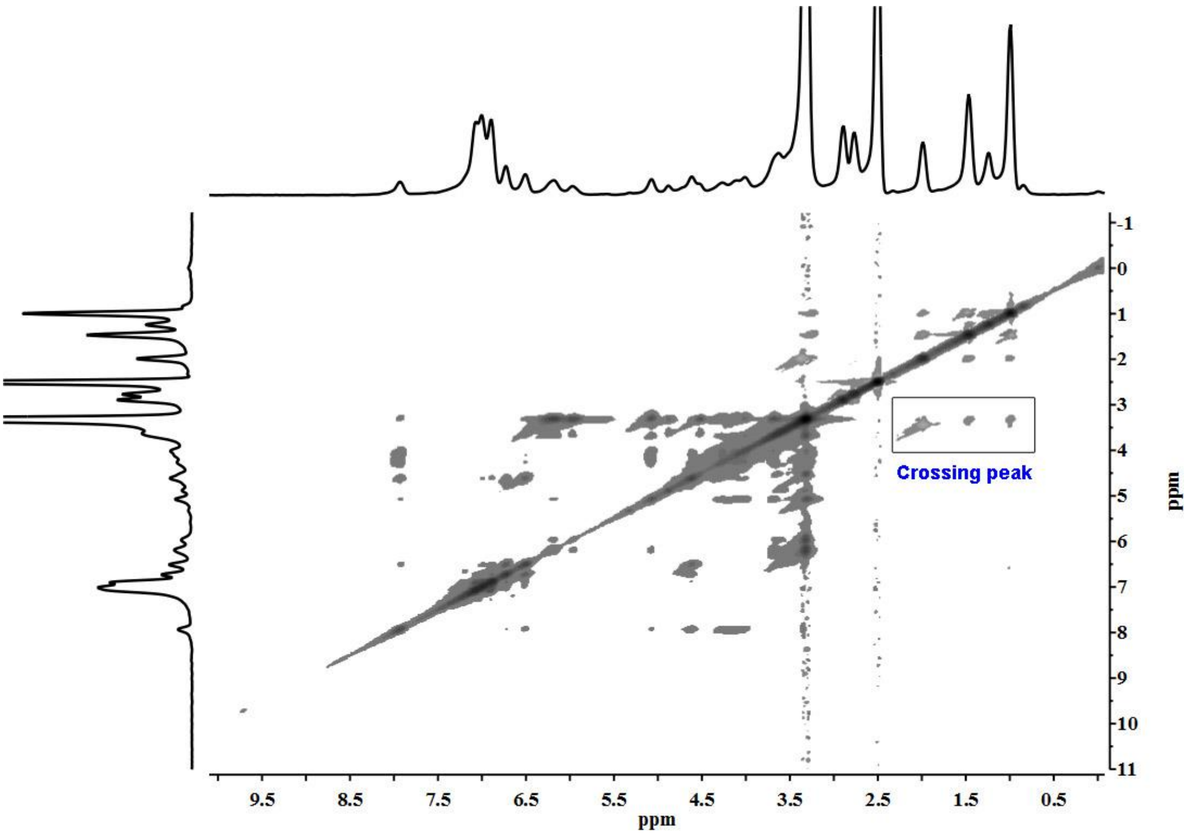

Figure S10. 2D NOESY ^{1}H NMR of SCAIECD and **1** in DMSO.

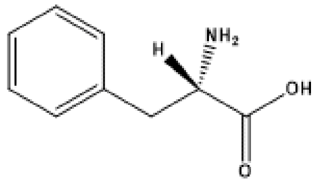

A

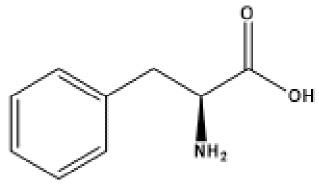

B

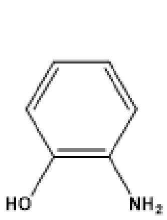

C

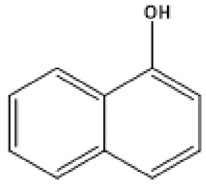

D

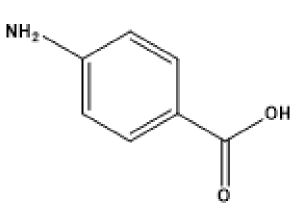

E

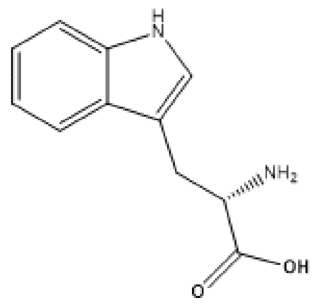

F

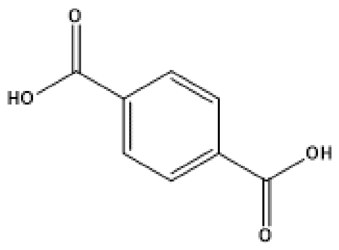

G

Figure S11. Structure of some interfering substances. **A**: L-Phenylalanine; **B**: D-Phenylalanine; **C**: 2-Aminophenol; **D**: 1-naphthalenol; **E**: 4-aminobenzoic acid; **F**: L-Tryptophane; **G**: p-phthalic acid.

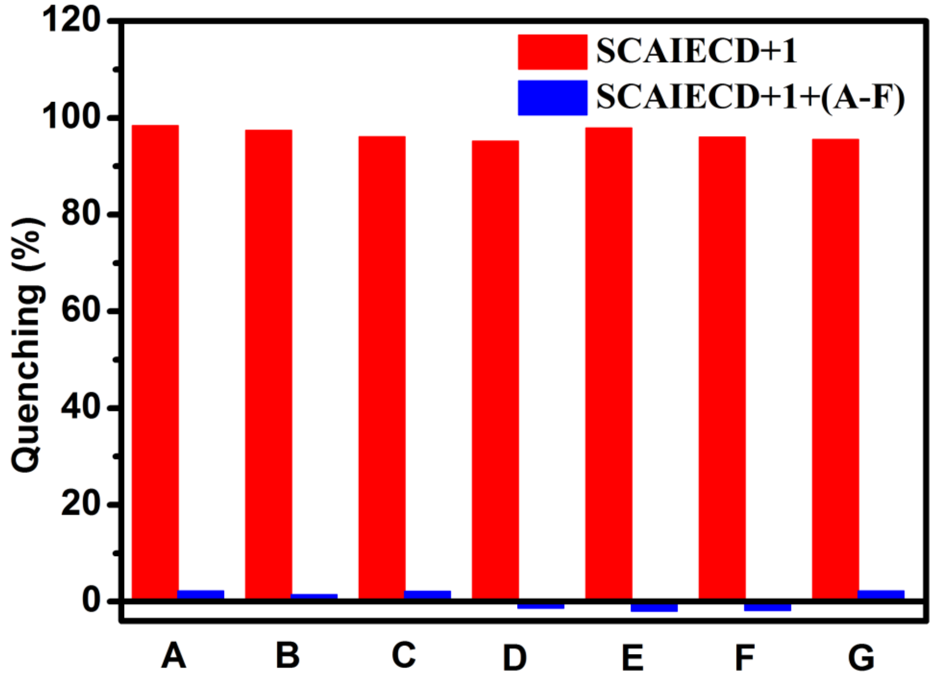

Figure S12. Percentage fluorescence quenching of SCAIECD (5 μM) with some interfering substances (A–F) and the hydrogenase of 1 (red bars). Percentage fluorescence quenching of SCAIECD (5 μM) only in the presence of some interfering substances (A-F) (blue bars).
